# Oral small molecule agents in management of ulcerative colitis: fact or fancy?

**DOI:** 10.55730/1300-0144.5722

**Published:** 2023-08-11

**Authors:** Benan KASAPOĞLU, Atilla ERTAN

**Affiliations:** 1 Division of Gastroenterology, Department of Internal Medicine, Faculty of Medicine, Lokman Hekim University, Ankara, Turkiye; 2 Division of Gastroenterology, Department of Internal Medicine, Faculty of Medicine, University Texas McGovern Medical School, Houston, TX, USA

**Keywords:** Ulcerative colitis, sphingosine-1-phosphate receptor modulators, tofacitinib, upadacitinib, ozanimod

## Abstract

Ulcerative colitis is a chronic, immune-mediated disease characterized by recurring episodes of mucosal inflammation in the colon and rectum. The primary pathogenic mechanism of ulcerative colitis is the dysregulation of the mucosal immune response. The disease follows a relapsing-remitting course, and the goal of management is to successfully induce and then maintain remission. Effectively managing this chronic disease requires addressing all aspects of it. Currently, we have various antitumor necrosis factor agents and novel biologics available for treating ulcerative colitis patients with moderate-to-severe disease. However, none of the existing treatments are considered entirely satisfactory or ideal in these cases. After extensive progressive research, oral small molecule therapies targeting mediators of ongoing inflammation represent an exciting and revolutionary change in the treatment of ulcerative colitis, especially for patients with moderate-to-severe disease. In this review, we aimed to summarize the available experience and ongoing research on oral small molecule agents in the management of ulcerative colitis. The available experience and ongoing research with promising outcomes provide convincing evidence that the value of oral small molecule agents is fact not fancy.

## 1. Introduction

Ulcerative colitis (UC) is a chronic, immune-mediated disease characterized by recurrent episodes of mucosal inflammation in the colon and rectum. The primary pathogenic mechanism of UC is the dysregulation of the mucosal immune response. The disease follows a relapsing-remitting course, and the goal of management is to successfully induce and then maintain remission. Effectively managing this disease requires addressing every aspect of it, necessitating long-term follow-up.The pathogenesis of UC is a complex process involving both the innate and adaptive immune systems. Neutrophils act as the “first responders” in this process, and following their initial response, innate inflammatory neutrophils and monocytes create an inflammatory environment with the assistance of proinflammatory cytokines such as the interleukin (IL)-1 family, IL-6, and tumor necrosis factor-α (TNF-α) [1]. On the other hand, regarding adaptive immunity, abnormal antigen(s) play a vital role in activating the aberrant T-cell response and creating a pathologic cytokine environment [2]. UC is associated with a Th2 response through elevated IL-4, IL-5, and IL-13 levels. However, recently, IL-23, which amplifies Th17 cell responses, has been identified as a key driver of the inflammatory response in UC [3]. Stimulation of IL-23 triggers the JAK-STAT signaling cascade, which sustains the production of proinflammatory cytokines through the activation of Th17 cells, such as IL6, IL17, IL21, IL22, and TNF-α [4].

Ulcerative colitis is an insidious disease that can impact various aspects of patients’ lives, including their functional and mental well-being. Therefore, the optimal management of this chronic disease must comprehensively address all its facets. While sulfasalazine, 5-aminosalicylates (5-ASA), corticosteroids, thiopurines, and methotrexate have long been considered the mainstay and first-line therapy for UC patients, biological agents are recommended for those with moderate-to-severe UC [5, 6]. Currently, we have various antitumor necrosis factor (anti-TNF) agents and novel biologics used in the treatment of UC for patients with moderate-to-severe disease. However, none of the available treatment methods are considered entirely satisfactory in these cases. The ideal treatment should be cost-effective, easy to manage, and reduce the need for hospitalizations while also preventing long-term complications, including the development of colorectal cancer. Although anti-TNF agents and novel biologics may achieve normal bowel function and a good quality of life with successful induction of long-term clinical remission, approximately half of the moderate-to-severe UC cases require additional management options. Therapeutic strategies for these patients are evolving. After extensive and progressive research, oral small molecule therapies targeting mediators of active intestinal inflammation sources represent an exciting and revolutionary advancement in UC treatment [7].

In this review, we aimed to summarize the available experience and ongoing research on oral small molecule agents in the management of UC patients.

## 2. Oral small molecule agents

### 2.1. Janus kinase [JAK] and/or tyrosine kinase 2 inhibitors

Janus kinase (JAK) proteins are intracellular cytoplasmic tyrosine kinases, and the Janus kinase–signal transducer and activator of transcription (JAK-STAT) pathway plays an essential role in various cellular processes contributing to both innate and adaptive immunity, which are also involved in the pathogenesis of UC. Initially, an extracellular ligand binds to cytokine receptors, activating JAKs through phosphorylation, which then transmits inflammation by activating STAT transcription factors. This pathway is crucial in the differentiation, migration, and survival of T lymphocytes. Inhibition of different JAK subtypes will have diverse effects by regulating different cytokine receptors [8,9]. Therefore, blocking the JAK-STAT pathway can intercept numerous cytokine signals, making it a potential therapeutic approach for many immune-mediated inflammatory diseases [10]. There are four subtypes of JAK proteins defined as JAK1, JAK2, JAK3, and TYK2.

Similarly, in UC, the inflammatory response is primarily characterized by helper T-cell subtype 2, which triggers the production of proinflammatory cytokines by initiating the JAK-STAT signaling cascade [3,4]. Janus kinase inhibitors represent an entirely new category of treatment for immune-mediated inflammatory diseases, such as UC. Since these agents directly target JAK-dependent cytokines, they can regulate the response of many proinflammatory cytokines involved in the pathogenesis of UC. They offer several advantages, including oral administration, rapid onset of action, and short half-lives [11]. Initially, the JAK1/3 inhibitor tofacitinib has shown effectiveness, and currently, there are other anti-JAK inhibitors under investigation for managing UC patients (Table 1) [12–15]. Both tofacitinib and upadacitinib are approved by the US Food and Drug Administration (FDA) for treating moderate-to-severe UC patients.

#### 2.1.a. Tofacitinib

**Figure 1:**
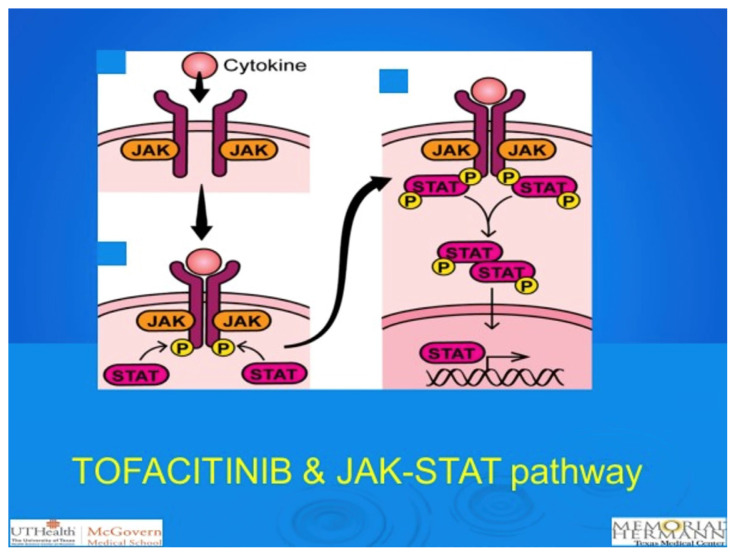
Janus kinase (JAK) proteins are intracellular cytoplasmic tyrosine kinases and Janus kinase–signal transducer and activator of transcription (JAK-STAT) pathway.

Tofacitinib is a fast-acting oral JAK inhibitor with clinical effects observed within days of induction therapy. It primarily targets JAK1 and JAK3 (Figure 1). Recently, in two phase 3 induction studies (Octave) over 8 weeks, the efficacy and safety of tofacitinib were investigated. At the Ertan Digestive Disease Center, we have also participated in both the induction and phase 3 maintenance studies (Octave studies) of tofacitinib in UC patients with moderate-to-severe disease. In both the Octave induction trials and the Octave-Sustain trial, significantly higher rates of clinical remission were achieved compared to the placebo groups at 8 and 52 weeks, respectively. However, rates of overall infection, herpes zoster infection, and thromboembolic events were more common with tofacitinib than with the placebo group [16]. Colombel et al. [17] reported that 77.5% of patients had a clinical response with tofacitinib 10 mg daily at 12 months, and that 56.0% had a clinical response without any additional identified safety risks at 36 months. A recent metaanalysis [18] demonstrated that tofacitinib was successful in refractory UC patients with a tolerable safety profile. In another recent case-control study, in biologic-experienced hospitalized patients with acute severe UC, tofacitinib was found to be an effective treatment method in remission induction when combined with i.v. corticosteroids [19]. In a very recent study comparing vedolizumab and tofacitinib in anti-TNF refractory UC patients, tofacitinib was reported to be more effective than vedolizumab with similar safety outcomes [20]. With its rapid action, oral administration, positive results in induction and maintenance studies, and a manageable safety profile, tofacitinib is a promising agent in the treatment of UC patients. Further comparison of tofacitinib with various biological agents for the induction and maintenance of UC is needed.

#### 2.1.b. Peficitinib

Peficitinib is an oral pan-JAK inhibitor with moderate selectivity for JAK3. It was approved for the treatment of another immune-mediated disease, rheumatoid arthritis, in patients unresponsive to standard therapy [21]. In a phase 2b dose-ranging trial in ulcerative colitis patients, peficitinib did not achieve the desired outcome, and further studies with higher doses are pending for UC patients [22]. Despite the initial phase 2b study not yielding the desired results, considering its mechanism of action, peficitinib still holds promise as a potential agent in the treatment of UC.

#### 2.1.c. Filgotinib

Filgotinib is a selective JAK1 inhibitor with a half-life of 6 h [23]. In the phase 2b/3 Selection trial conducted in multiple centers, including the Ertan Digestive Disease Center, filgotinib at a dose of 200 mg successfully induced clinical remission by week 10 and maintained it through week 58 compared to placebo in patients with moderate-to-severe UC, with a serious adverse event rate of less than 5% [24]. In post hoc analyses of the Selection trial, filgotinib at a daily dose of 200 mg demonstrated rapid (starting on day 7) and sustained improvements in both ulcerative colitis symptoms and quality of life associated with health by week 58 [25].

In a recent network metaanalysis of seven randomized controlled trials involving 3190 patients, filgotinib at a dose of 100 mg was determined to be the most effective option for inducing endoscopic remission. On the other hand, in the overall assessment of mucosal healing, clinical remission, and alterations in Mayo score, tofacitinib at a dose of 3 mg was identified as the preferred treatment option. Fortunately, adverse events and treatment discontinuation rates were similar between the JAK inhibitors and placebo groups [26]. With its rapid clinical and endoscopic response, coupled with a low side-effect profile, filgotinib appears to hold promise in the treatment of UC.

#### 2.1.d. Upadacitinib

Upadacitinib is an oral selective JAK1 inhibitor with a half-life of 4 h [27]. In a multicenter phase 2b study, upadacitinib demonstrated greater efficacy compared to placebo in inducing remission by week 8 in UC patients. However, in that induction study, upadacitinib led to elevations in serum lipid levels and creatine phosphokinase [28]. In a phase 3 study, significantly higher rates of clinical remission were achieved with upadacitinib at a dose of 45 mg compared to the placebo group. Similarly, in the maintenance study, by week 52, clinical remission was established at a significantly higher rate in the upadacitinib group [29]. The most common adverse events included elevations in creatine phosphokinase, nasopharyngitis, and acne. In a recent systematic review of 29 studies, upadacitinib emerged as the most effective treatment modality for inducing clinical remission, with no significant differences observed between treatment modalities regarding adverse events [30]. In a study involving 988 patients, upadacitinib treatment resulted in significant improvements in all UC symptoms compared to placebo from the initial days of treatment, and these improvements were sustained for 2 weeks [31]. Like other JAK inhibitors, upadacitinib proves highly effective in both inducing and maintaining remission in UC treatment. While some adverse events, such as elevations in creatine phosphokinase and nasopharyngitis, were reported during remission induction with this treatment, there were no serious adverse events or deaths related to the treatment in the maintenance study. With its rapid action and favorable safety profile, upadacitinib also stands as a promising agent for UC patients.

#### 2.1.e. Itacitinib

Itacitinib is a selective JAK1 inhibitor [8, 32]. Regarding the mechanism of action, like other JAK inhibitors, itacitinib holds promise for the future in the treatment of immune-mediated diseases. In an acute graft-versus-host disease model, treatment with itacitinib led to a prompt and significant decrease in inflammatory mediators within both lymphocytes and target tissue, resulting in a noticeable improvement in disease symptoms. The preliminary research trial with this agent is pending in inflammatory bowel diseases.

#### 2.1.f. Ritlecitinib and Brepocitinib

Ritlecitinib (an oral JAK3/TEC inhibitor) and brepocitinib (an oral TYK2/JAK1 inhibitor) are dual inhibitors of the JAK/STAT pathway, and their efficacy and safety have been studied in various immune-mediated inflammatory diseases [10, 33]. In induction studies, involving 319 randomized patients with moderate-to-severe UC, ritlecitinib and brepocitinib were reported to be more efficient than placebo within 8 weeks [34–36]. Further studies, especially focusing on the maintenance of treatment, are warranted for ritlecitinib and brepocitinib.

#### 2.1.g. Deucravacitinib

Unlike JAK 1-3 inhibitors, deucravacitinib (previously known as BMS-986165) is a novel, oral, highly selective TYK2 inhibitor. Deucravacitinib binds to the regulatory (JH2 pseudo-kinase) domain and inhibits its interaction with the catalytic domain. This, in turn, leads to the inactivation of TYK2, effectively stopping signal transduction [9, 37]. Deucravacitinib exhibits little to no activity against JAK 1-3. In a double-blind phase 2 study (LATTICE-UC), deucravacitinib (6 mg twice daily) did not achieve a better clinical or endoscopic response than placebo at week 12 [38]. However, a higher dose of deucravacitinib will be assessed in UC patients.

### 2.2. Sphingosine-1-phosphate receptor modulators

**Figure 2:**
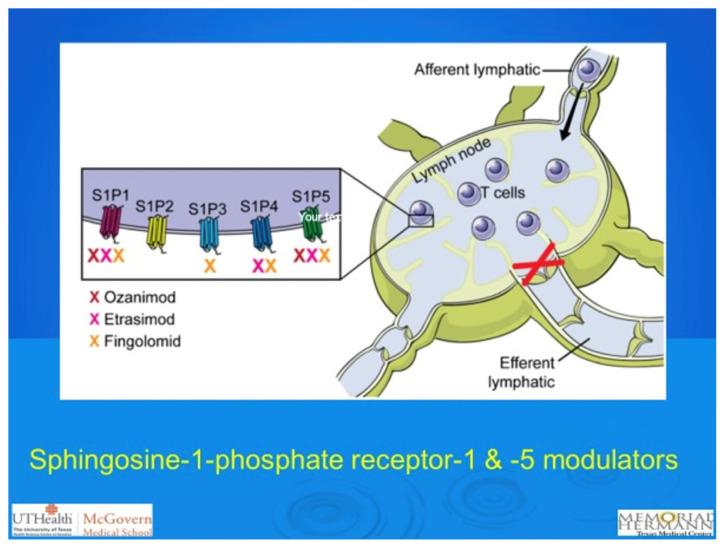
The sphingosine-1-phosphate (S1P) receptors in lymph nodes.

The sphingosine-1-phosphate (S1P) receptors constitute a family of five receptors (S1P1-S1P5) that regulate numerous immunologic and cardiovascular effects (Figure 2). Especially, S1P1 has been shown to play a vital role in the trafficking of lymphocytes from lymphoid organs, reducing their infiltration [39]. For this reason, S1P receptors have been identified as promising targets in the treatment of immune-mediated diseases (Table 2). The FDA has already approved ozanimod for the treatment of patients with moderate-to-severe UC.

#### 2.2.a. Ozanimod

Ozanimod (RPC1063) is an oral agonist of the S1P1 and S1P5 receptors, encouraging peripheral lymphocyte sequestration, thereby reducing the movement of activated lymphocytes to the gastrointestinal tract [40, 41]. By decreasing lymphocyte migration into the inflamed intestine, ozanimod has been identified as an effective agent in the treatment of UC patients [42]. However, it is important to note that since S1P signaling is also involved in cardiovascular functions and heart rate, S1PR1 modulators may lead to bradycardia and atrioventricular block [43].

In the Touchstone trial (n=197), ozanimod once a day was found to be effective in UC treatment during both the induction (8 weeks) and maintenance periods (24 weeks). Furthermore, the long-term efficacy of ozanimod treatment was supported by the open-label extension results of the study. In the 10-week induction and the 52-week maintenance periods, ozanimod resulted in significantly higher rates of clinical remission compared to the placebo in patients with moderate-to-severe UC. Serious infections occurred at a proportion similar to the placebo during the 52-week trial. The ozanimod group had a higher frequency of mild elevations in liver aminotransferase levels. This trial was based on the 7-day dose-escalation schedule, which may be the reason for the absence of clinically significant bradycardia or cardiac conduction abnormalities in this study. Opportunistic infections and macular edema were observed in the ozanimod group, but with a very low incidence [44, 45].

Ozanimod is generally well tolerated in phase 2 and 3 trials. The main adverse events related to ozanimod mainly include upper respiratory tract infections, liver enzyme elevations, headache, pyrexia, nausea, and arthralgia. However, these side effects were not severe enough to lead to drug discontinuation [42, 45]. Considering all these results, Ozanimod can be suggested as an effective treatment agent in both induction and maintenance therapies for UC patients, with a good safety profile.

#### 2.2.b. Etrasimod and amiselimod

Etrasimod and amiselimod are other oral selective S1P1 receptor modulators. In a phase 2 study (Oasis), etrasimod 2 mg treatment resulted in significantly better improvement in modified Mayo Clinic scores than placebo at the 12th week of treatment, with only mild adverse events [46]. In an open-label extension of Oasis for 52 weeks, etrasimod 2 mg established a good safety profile in UC patients [47]. In the treatment of ulcerative colitis, etrasimod showed promising results with its rapid effect and only mild adverse effects.

In an experimental model of chronic colitis, the number of infiltrating T-helper cells into the colon was significantly decreased in the amiselimod-administered group, which inhibited the development of chronic colitis [48]. Considering the pathophysiological aspect, etrasimod and amiselimod could be good treatment alternatives for remission induction and maintenance in UC patients; however, further long-term results of maintenance studies with these agents are warranted.

#### 2.2.c. Fingolimod

Fingolimod is an agonist of four S1P receptor subtypes (S1P1, S1P3, S1P4, and S1P5) [49]. The effects of fingolimod in autoimmune diseases were first defined in Multiple Sclerosis [50]. S1P1 is suggested to be essential in preserving colonic vascular integrity and has been shown to be overexpressed in the colonic mucosa of UC patients [51]. In experimental models, fingolimod exhibited encouraging results in inflammatory bowel diseases. However, some adverse events such as atrioventricular block, bradycardia, and liver enzyme elevations were also reported [52–54]. In terms of the mechanism of action, fingolimod may be a hopeful agent in ulcerative colitis treatment; however, clinicians should be aware of the potential adverse events. For this reason, selective S1P modulators may offer a better balance of efficacy and safety in UC treatment.

### 2.3. Oral integrin inhibitors

The main driver of the pathogenesis of UC is the migration of lymphocytes to the gut mucosa. Chemokines and selectins are the chief mediators in this process, facilitating the adhesion of T cells to the endothelial cells. Novel treatment modalities have been developed for inflammatory bowel diseases, selectively targeting adhesion molecules like AJM 300, PN-943, and MORF-057 [55].

#### 2.3.a. AJM300

As an antagonist of the α4 integrin subunit, AJM300 prevents the binding of alpha-4 beta-7 (α4β7) and alpha-4 beta-1 integrin on T cells to adhesion molecules, inhibiting the migration of lymphocytes into the gut.

In a double-blind phase 2a study and in the phase 3 trial, AJM300 (960 mg three times daily) was found to improve endoscopic and clinical response at week 8 in UC patients with active disease, without any serious adverse events [56]. Although, theoretically, AJM300 could pose an augmented risk of progressive multifocal leukoencephalopathy (PML) due to its potential to reduce lymphocyte trafficking to the brain, no PML cases have been reported with the use of AJM300.

Based on this data, AJM300 could be a new treatment option for inducing remission in moderately active UC patients after further convincing studies, especially with long-term results.

#### 2.3.b. PN-943 and MORF-057

PN-943 is an oral small molecule gut-restricted agent that inhibits the alpha-4 beta-7 receptor as an integrin antagonist. In a recent 12-week, double-blind, placebo-controlled multicenter phase 2 trial [Ideal study], a better clinical remission with PN-943 compared to placebo with minimal adverse events was reported in UC patients [57]. The phase 3 trial is pending with this promising, oral small molecule agent.

MORF-057 is another oral small molecule gut-restricted agent that inhibits the alpha-4 beta-7 receptor. It was used in the Emerald-1 phase 1 study presented at the ACG meeting in 2022 with an exciting pharmacokinetic profile. Subsequently, a phase-2a research trial with this agent in patients with moderate-to-severe UC was presented at the United European Gastroenterology Week in 2022 [58]. A dose of 200 mg of MORF-057 was found to be safe and more effective than placebo in a 12-week induction study. A phase-2b study in UC treatment is pending [59].

### 2.4. Phosphodiesterases

There are two important intracellular second messengers: cyclic adenosine monophosphate (cAMP) and cyclic guanosine monophosphate (cGMP). These second messengers adjust numerous intracellular signal transduction pathways and regulate various physiological conditions such as cell proliferation, inflammation, and immune response. On the other hand, the degradation of cAMP and cGMP is catalyzed by phosphodiesterases (PDEs) [60]. In turn, the inhibition of PDEs is associated with the inhibition of inflammation.

#### 2.4.a. Apremilast

An oral PDE4 inhibitor, apremilast is being studied in inflammatory bowel diseases. PDE4 stimulates important inflammatory processes in neutrophils, including chemotaxis, degranulation, and adhesion to endothelial cells. For this reason, PDE4 inhibitors are considered potential suppressors of many inflammatory conditions [61, 62]. Even though the phase 2 trial with 30 mg or 40 mg apremilast did not meet the primary endpoint of clinical remission, clinical remission was sustained in 40% of 170 patients with moderate-to-severe UC [63]. The FDA has approved this agent for patients with psoriatic arthritis and Behcet’s disease.

### 2.5. Oral TNF agents

**Figure 3:**
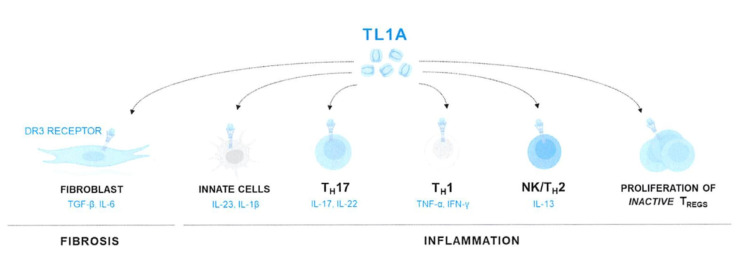
TL1A mediates both inflammation and fibrosis.

TNF-like factor 1A (TL1A) is a member of the tumor necrosis factor (TNF) family. Through its receptor, death receptor 3 (DR3), TL1A has been shown to influence multiple cell lineages (Figure 3). One prototype of TL1A, ABX464, is an oral small molecule that upregulates a single micro-RNA and down-regulates proinflammatory cytokines as well as TH17+ cells. This agent was effective with a daily dosage of one tablet (25 mg, 50 mg, and 100 mg doses) in 254 UC patients in clinical remission, performing much better than the placebo in the phase 2b trial with no major adverse events [64,65]. It is important to note that ABX464 also demonstrated rapid efficacy in UC patients refractory to biologics and/or tofacitinib treatment. The 2-year ABX464 open-label maintenance trial showed the long-term safety and tolerability of a 50 mg single dose. Research trials are pending in patients with Crohn’s disease, rheumatoid arthritis, as well as a phase 3 trial in patients with moderate-to-severe UC. If the phase 3 trial demonstrates similar effectiveness and tolerability as seen in phase 2, this agent may be a game-changer in the management of patients with IBD. Confirming TL1A’s antifibrinolytic effect in human trials would be tremendously important for patients with CD and beyond.

## 3. Conclusion

In the management of moderate-to-severe UC patients, despite the noteworthy improvements, novel treatment options are required due to remarkable rates of primary nonresponse, loss of response, and/or adverse events. Moreover, there is a high-cost burden associated with the intravenous administration or subcutaneous injections of biologics. For these reasons, researchers have been focusing on advances in oral treatments for chronic immune-mediated inflammatory conditions, such as ulcerative colitis, in recent years. Fortunately, the rapidly growing number of targeted therapies with oral small molecule agents offer advantages summarized in Table 3.

Therefore, new promising targets for the treatment of moderate-to-severe UC have emerged, which regulate several inflammatory and molecular pathways. There is a large number of promising therapies that make the future of patients with ulcerative colitis promising. Among these oral small molecules, selective JAK inhibitors have been shown to be effective alternatives with high rates of clinical remission. Gut-selective options of JAK inhibitors such as JAM 300, PM-943, MORF-057, or APX464 may potentially decrease systemic toxicities. Oral antiintegrin molecules may emerge as a novel option for the management of UC patients with moderate-to-severe disease. S1PR modulators offer lymphocyte entrapping in lymphoid organs and are potentially effective, particularly for ulcerative colitis.

In conclusion, a large number of promising therapies make the future of patients with moderate-to-severe ulcerative colitis promising. The available experience and ongoing research with promising outcomes are quite convincing that the value of oral small molecule agents is a fact, not fancy.

## Figures and Tables

**Table 1:** Oral janus kinase and/or tyrosine kinase 2 inhibitors in the treatment of ulcerative colitis.

	Mechanism of action	Notes	Adverse events
**Tofacitinib**	Pan-JAK inhibitor, especially JAK1 and JAK3	In Octave induction and Octave sustain studies; clinical remission rates of UC patients were significantly higher compared with the placebo groups.	Rare: The overall infection, herpes zoster infection, and thromboembolic event rates increase.
**Peficitinib**	Pan-JAK inhibitor (moderately selective for JAK3)	In phase 2b trial, peficitinib could not achieve a successful outcome in UC patients.	
**Filgotinib**	Selective JAK1 inhibitor	Filgotinib 200 mg/day was efficient in the induction and maintenance of clinical remission in UC patients (selection trials).	Rare: There were no significant differences between the filgotinib and placebo groups regarding the incidence of infections.
**Upadacitinib**	Selective JAK1 inhibitor	In remission induction and maintenance of UC, upadacitinib was effective.	Common: Creatine phosphokinase elevation, nasopharyngitis, and acne Rare: arthralgia, infections
**Itacitinib**	Selective JAK1 inhibitor	The preliminary research trial with this agent is pending for inflammatory bowel diseases.	Rare: diarrhea
**Ritlecitinib and brepocitinib**	Dual inhibitors of the JAK/STAT pathway	Ritlecitinib and brepocitinib were effective in the remission induction of UC.	Rare: Mild infections and rhabdomyolysis may occur during the brepocitinib treatment.
**Deucravacitinib**	Selective TYK2 inhibitor	Deucravacitinib (6 mg twice daily) could not achieve a better clinical or endoscopic response than the placebo at week 12. (LATTICE-UC)	Common: infections, headache, and rash

**Table 2:** Oral sphingosine-1-phosphate receptor modulators in the treatment of ulcerative colitis.

	Mechanism of action	Notes	Adverse events
**Ozanimod**	S1P1 and S1P5 receptor agonist	Once daily ozanimod was effective in both the induction of remission and maintenance of UC. (Touchstone trials)	Common: Upper respiratory tract infections, liver enzymes elevations, headache, pyrexia, nausea, and arthralgia.
**Etrasimod and amiselimod**	Selective S1P1 receptor agonists	Etrasimod 2 mg led to significantly better clinical improvements than placebo in the induction and maintenance of UC with a good safety profile. (Oasis studies) The number of infiltrating T-helper cells into the colon was significantly decreased with Amiselimod administration in an experimental model of chronic colitis.	Rare: Leukopenia, anemia, infections, transaminase elevation, and cardiovascular events.
**Fingolimod**	S1P1, S1P_3_, S1P_4_ , and S1P5 receptor agonist	In mouse models of inflammatory bowel disease, this drug was shown to exhibit encouraging results.	Rare: atrioventricular block, bradycardia, and liver enzyme elevations

**Table 3:** Advantages of oral small molecule agents in patients with ulcerative colitis.

Ease administration
Relatively cheaper
Predictable pharmacokinetic studies
Durable effectiveness comparable with biologics
Fast-on action and fast-off outcome
No immunogenicity
More effective in IBD patients with significant hypoalbuminemia
Potential for combination treatment with biologic agents

## References

[1] Friedrich M, Pohin M, Powrie F (2019). Cytokine Networks in the Pathophysiology of Inflammatory Bowel Disease. *Immunity*.

[2] Graham DB, Luo C, O’Connell DJ, Lefkovith A, Brown EM (2018). Antigen discovery and specification of immunodominance hierarchies for MHCII-restricted epitopes. *Nature Medicine*.

[3] Porter RJ, Kalla R, Ho GT (2020). Ulcerative colitis: Recent advances in the understanding of disease pathogenesis. F1000 Research.

[4] Noviello D, Mager R, Roda G, Borroni RG, Fiorino G (2021). The IL23-IL17 Immune Axis in the Treatment of Ulcerative Colitis: Successes, Defeats, and Ongoing Challenges. Frontiers in Immunology.

[5] Le Berre C, Roda G, Nedeljkovic Protic M, Danese S, Peyrin-Biroulet L (2020). Modern use of 5-aminosalicylic acid compounds for ulcerative colitis. Expert Opinion on Biological Therapy.

[6] Chhibba T, Ma C (2020). Is there room for immunomodulators in ulcerative colitis?. Expert Opinion on Biological Therapy.

[7] Herman A, Ertan A (2022). Oral Agents in the Treatment of Inflammatory Bowel Disease: A Remarkable Progress. *Current* Trends in *Gastroenterology* and *Hepatology*.

[8] Burke JR, Cheng L, Gillooly KM, Strnad J, Zupa-Fernandez A (2019). Autoimmune pathways in mice and humans are blocked by pharmacological stabilization of the TYK2 pseudokinase domain. Science Translational Medicine.

[9] Danese S, Peyrin-Biroulet L (2021). Selective Tyrosine Kinase 2 Inhibition for Treatment of Inflammatory Bowel Disease: New Hope on the Rise. Inflammatory Bowel Diseases.

[10] Covington M, He X, Scuron M, Li J, Collins R (2020). Preclinical characterization of itacitinib (INCB039110), a novel selective inhibitor of JAK1, for the treatment of inflammatory diseases. European Journal of Pharmacology.

[11] Lefevre PLC, Vande Casteele N (2020). Clinical pharmacology of janus kinase inhibitors in inflammatory bowel disease. Journal of Crohn’s and Colitis.

[12] Banerjee S, Biehl A, Gadina M, Hasni S, Schwartz DM (2017). JAK-STAT signaling as a target for inflammatory and autoimmune diseases: current and future prospects. *Drugs*.

[13] Kerschbaumer A, Smolen JS, Nash P, Doerner T, Dougados M (2020). Points to consider for the treatment of immune-mediated inflammatory diseases with Janus kinase inhibitors: a systematic literature research. Rheumatic Journal-Musculoskeletal Disorders Open.

[14] Ferrante M, Sabino J (2020). Efficacy of JAK inhibitors in Ulcerative Colitis. Journal of Crohn’s and Colitis.

[15] Ma C, Lee JK, Mitra AR, Teriaky A, Choudhary D (2019). Systematic review with meta-analysis: efficacy and safety of oral Janus kinase inhibitors for inflammatory bowel disease. Alimentary *Pharmacology* & *Therapeutics*.

[16] Sandborn WJ, Su C, Panes J (2017). Tofacitinib as Induction and Maintenance Therapy for Ulcerative Colitis. *New England Journal of Medicine*.

[17] Colombel JF, Osterman MT, Thorpe AJ, Salese L, Nduaka CI (2022). Maintenance of Remission With Tofacitinib Therapy in Patients With Ulcerative Colitis. Clinical Gastroenterology and Hepatology.

[18] Taxonera C, Olivares D, Alba C (2022). Real-World Effectiveness and Safety of Tofacitinib in Patients with Ulcerative Colitis: Systematic Review with Meta-Analysis. Inflammatory Bowel Diseases.

[19] Berinstein JA, Sheehan JL, Dias M, Berinstein EM, Steiner CA (2021). Tofacitinib for Biologic-Experienced Hospitalized Patients With Acute Severe Ulcerative Colitis: A Retrospective Case-Control Study. Clinical Gastroenterology and Hepatology.

[20] Straatmijer T, Biemans VBC, Visschedijk M, Hoentjen F, de Vries A (2023). Initiative on Crohn and Colitis. Superior Effectiveness of Tofacitinib Compared to Vedolizumab in Anti-TNF-experienced Ulcerative Colitis Patients: A Nationwide Dutch Registry Study. Clinical Gastroenterology and Hepatology.

[21] Takeuchi T, Tanaka Y, Iwasaki M, Ishikura H, Saeki S (2016). Efficacy and safety of the oral Janus kinase inhibitor peficitinib (ASP015K) monotherapy in patients with moderate to severe rheumatoid arthritis in Japan: a 12-week, randomised, double-blind, placebo-controlled phase IIb study. Annals of the Rheumatic Diseases.

[22] Sands BE, WJ, Feagan BG, Lichtenstein GR, Zhang H (2018). Peficitinib-UC Study Group. Peficitinib, an Oral Janus Kinase Inhibitor, in Moderate-to-severe Ulcerative Colitis: Results from a Randomized, Phase 2 Study. Journal of Crohn’s and Colitis.

[23] Harris C, Cummings JRF (2021). JAK1 inhibition and inflammatory bowel disease. Rheumatology (Oxford).

[24] Feagan BG, Danese S, Loftus EV, Vermeire S, Schreiber S (2021). Filgotinib as induction and maintenance therapy for ulcerative colitis (SELECTION): a phase 2b/3 double-blind, randomized, placebo-controlled trial. Lancet.

[25] Danese S, Ferrante M, Feagan BG, Peyrin-Biroulet L, Hibi T (2023). Rapid and Sustained Symptom Relief in Patients with Ulcerative Colitis Treated With Filgotinib: Data From the Phase 2b/3 SELECTION Trial. American Journal of Gastroenterology.

[26] Li Y, Yao C, Xiong Q, Xie F, Luo L (2022). Network meta-analysis on efficacy and safety of different Janus kinase inhibitors for ulcerative colitis. *Journal* of *Clinical Pharmacy* and *Therapeutics*.

[27] Mohamed MF, Camp HS, Jiang P, Padley RJ, Asatryan A (2016). Pharmacokinetics, safety and tolerability of ABT-494, a novel selective JAK 1 inhibitor, in healthy volunteers and subjects with rheumatoid arthritis. *Clinical Pharmacokinetics*.

[28] Sandborn WJ, Ghosh S, Panes J, Schreiber S, D’Haens G (2020). Efficacy of Upadacitinib in a Randomized Trial of Patients With Active Ulcerative Colitis. Gastroenterology.

[29] Danese S, Vermeire S, Zhou W, Pangan AL, Siffledeen J (2022). Upadacitinib as induction and maintenance therapy for moderately to severely active ulcerative colitis: results from three phase 3, multicentre, double-blind, randomised trials. Lancet.

[30] Lasa JS, Olivera PA, Danese S, Peyrin-Biroulet L (2022). Efficacy and safety of biologics and small molecule drugs for patients with moderate-to-severe ulcerative colitis: a systematic review and network meta-analysis. Lancet Gastroenterology and Hepatology.

[31] Loftus EV, Colombel JF, Takeuchi K, Gao X, Panaccione R (2022). Upadacitinib Therapy Reduces Ulcerative Colitis Symptoms as Early as Day 1 of InductionTreatment. Clinical Gastroenterology and Hepatology.

[32] Courtois J, Ritacco C, Dubois S, Canti L, Vandenhove B (2021). Itacitinib prevents xenogeneic GVHD in humanized mice. Bone Marrow Transplantation.

[33] Fensome A, Ambler CM, Arnold E, Banker ME, Brown MF (2018). Dual inhibition of TYK2 and JAK1 for the treatment of autoimmune diseases: discovery of ((S)-2,2-difluorocyclopropyl)((1 R,5 S)-3-(2-((1-methyl-1 H-pyrazol-4-yl)amino)pyrimidin-4-yl)-3,8-diazabicyclo[3.2.1] octan-8-yl) methanone (PF-06700841). *Journal of Medicinal Chemistry*.

[34] Winnette R, Banerjee A, Sikirica V, Peeva E, Wyrwich K (2022). Characterizing the relationships between patient-reported outcomes and clinician assessments of alopecia areata in a phase 2a randomized trial of ritlecitinib and brepocitinib. Journal of the European Academy of Dermatology and Venereology.

[35] Sandborn WJ, Danese S, Leszczyszyn J, Romatowski J, Altintas E (2023). Oral Ritlecitinib and Brepocitinib for Moderate-to-Severe Ulcerative Colitis: Results From a Randomized, Phase 2b Study. Clinical Gastroenterology and Hepatology.

[36] Wrobleski ST, Moslin R, Lin S, Wrobleski ST, Moslin R (2019). Highly selective inhibition of tyrosine kinase 2 (TYK2) for the treatment of autoimmune diseases: discovery of the allosteric inhibitor BMS-986165. Journal of Medicinal Chemistry.

[37] Moslin R, Zhang Y, Wrobleski ST, Lin S, Mertzman M (2019). Identification of N-methyl nicotinamide and N-methyl pyridazine-3-carboxamide pseudokinase domain ligands as highly selective allosteric inhibitors of tyrosine kinase 2 (TYK2). Journal of Medicinal Chemistry.

[38] (2022). Efficacy and Safety of Deucravacitinib, an Oral, Selective Tyrosine Kinase 2 Inhibitor, in Patients With Moderately to Severely Active Ulcerative Colitis: 12-Week Results From the Phase 2 LATTICE-UC Study. Gastroenterology and Hepatology (N Y).

[39] Schwab SR, Cyster JG (2007). Finding a way out: lymphocyte egress from lymphoid organs. Nature Immunology.

[40] Scott FL, Clemons B, Brooks J, Brahmachary E, Powell R (2016). Ozanimod (RPC1063) is a potent sphingosine-1-phosphate receptor-1 (S1P1) and receptor-5 (S1P5) agonist with autoimmune disease-modifying activity. British Journal of Pharmacology.

[41] Paik J (2022). Ozanimod: A Review in Ulcerative Colitis. Drugs.

[42] Bristol Myers Squibb EEIG Pharma (2021). Zeposia^®^ (ozanimod hydrochloride): EU summary of product characteristics.

[43] Jozefczuk E, Guzik TJ, Siedlinski M (2020). Significance of sphingosine-1-phosphate in cardiovascular physiology and pathology. Pharmacological Research.

[44] Sandborn WJ, Feagan BG, Wolf DC, D’Haens G, Vermeire S, TOUCHSTONE Study Group. (2016). Ozanimod Induction and Maintenance Treatment for Ulcerative Colitis New England. Journal of Medicine.

[45] Sandborn WJ, Feagan BG, D’Haens G, Wolf DC, Jovanovic I, True North Study Group (2021). Ozanimod as Induction and Maintenance Therapy for Ulcerative Colitis. New England Journal of Medicine.

[46] Sandborn WJ, Peyrin-Biroulet L, Zhang J, Chiorean M, Vermeire S (2020). Efficacy and Safety of Etrasimod in a Phase 2 Randomized Trial of Patients With Ulcerative Colitis. Gastroenterology.

[47] Vermeire S, Chiorean M, Panés J, Peyrin-Biroulet L, Zhang J (2021). Long-term Safety and Efficacy of Etrasimod for Ulcerative Colitis: Results from the Open-label Extension of the OASIS Study. Journal of Crohn’s and Colitis.

[48] Shimano K, Maeda Y, Kataoka H, Murase M, Mochizuki S (2019). Amiselimod (MT-1303), a novel sphingosine 1-phosphate receptor-1 functional antagonist, inhibits progress of chronic colitis induced by transfer of CD4+CD45RB high T cells. PLoS One.

[49] Billich A, Bornancin F, Devay P, Mechtcheriakova D, Urtz N (2003). Phosphorylation of the immunomodulatory drug FTY720 by sphingosine kinases. Journal of Biological Chemistry.

[50] Chun J, Hartung HP (2010). Mechanism of action of oral fingolimod (FTY720) in multiple sclerosis. Clinical Neuropharmacology.

[51] Montrose DC, Scherl EJ, Bosworth BP, Zhou XK, Jung B (2013). S1P_1_ localizes to the colonic vasculature in ulcerative colitis and maintains blood vessel integrity. Journal of Lipid Research.

[52] Daniel C, Sartory N, Zahn N, Geisslinger G, Radeke HH (2007). FTY720 ameliorates Th1-mediated colitis in mice by directly affecting the functional activity of CD4+CD25+ regulatory T cells. Journal of Immunology.

[53] Vargas WS, Perumal JS (2013). Fingolimod and cardiac risk: Latest findings and clinical implications. Therapeutic Advances in Drug Safety.

[54] Dal Buono A, Gabbiadini R, Alfarone L, Solitano V, Repici A (2022). Sphingosine 1-Phosphate Modulation in Inflammatory Bowel Diseases: Keeping Lymphocytes Out of the Intestine. Biomedicines.

[55] Chudy-Onwugaje KO, Christian KE, Farraye FA, Cross RK (2019). A State-of-the-Art Review of New and Emerging Therapies for the Treatment of IBD. Inflammatory Bowel Diseases.

[56] Sandborn WJ, Mattheakis LC, Modi NB, Pugatch D, Bressler B (2021). PTG-100, an Oral α4β7 Antagonist Peptide: Preclinical Development and Phase 1 and 2a Studies in Ulcerative Colitis. Gastroenterology.

[57] (2016). Safety and Efficacy Study of PTG-100 in the Treatment of Moderate to Severe Ulcerative Colitis Identifier NCT02895100.

[58] (2022). A Phase 2a Open-Label Study to Evaluate the Efficacy and Safety of MORF-057 in Adults With UC Identifier NCT05291689.

[59] (2022). A Study to Evaluate MORF-057 in Adults with Moderately to Severely Active UC (EMERALD-2) Identifier NCT05611671.

[60] Matsuoka K, Watanabe M, Ohmori T, Nakajima K, Ishida T, AJM300 Study Group (2022). AJM300 (carotegrast methyl), an oral antagonist of α4-integrin, as induction therapy for patients with moderately active ulcerative colitis: a multicentre, randomized, double-blind, placebo-controlled, phase 3 study. Lancet Gastroenterology and Hepatology.

[61] Ahmad F, Murata T, Shimizu K, Degerman E, Maurice D (2015). Cyclic nucleotide phosphodiesterases: Important signaling modulators and therapeutic targets. Oral Diseases.

[62] Schafer PH, Parton A, Capone L, Cedzik D, Brady H (2014). Apremilast is a selective PDE4 inhibitor with regulatory effects on innate immunity. Cell Signaling.

[63] Danese S, Neurath MF, Kopon A, Zhan X, Usuiskin K (2020). Effect of apremilast, an oral inhibitor of phosphodiesterase 4, in a randomized trial patients with active ulcerative colitis. Clinical Gastrenterology and Hepatology.

[64] Richard AC, Ferdinand JR, Meylan F, Hayes ET, Gabay O (2015). The TNF-family cytokine TL1A: from lymphocyte costimulator to disease co-conspirator. Journal of Leukocyte Biology.

[65] Vermeire S, Sands BE, Tilg H, Tulassay Z, Kempinski R (2020). ABX 464 (obefazimod) for moderate-to-severe, active ulcerative colitis: a phase 2b, double-blind, randomised, placebo-controlled induction trial a 48-week, open –label extension. The Lancet Gastroenterology & Hepatology.

